# Apricot Kernel Extract and Amygdalin Inhibit Urban Particulate Matter-Induced Keratoconjunctivitis Sicca

**DOI:** 10.3390/molecules24030650

**Published:** 2019-02-12

**Authors:** Soo-Wang Hyun, Junghyun Kim, Bongkyun Park, Kyuhyung Jo, Tae Gu Lee, Jin Sook Kim, Chan-Sik Kim

**Affiliations:** 1 Herbal Medicine Research Division, Korea Institute of Oriental Medicine, Daejeon 34054, Korea; swhyun@kiom.re.kr (S.-W.H.); jskim@kiom.re.kr (J.S.K.); 2 Department of Oral Pathology, School of Dentistry, Chonbuk National University, Jeonju 54896, Korea; dvmhyun@jbnu.ac.kr; 3 Clinical Medicine Division, Korea Institute of Oriental Medicine, Daejeon 34054, Korea; bkpark@kiom.re.kr (B.P.); jopd7414@kiom.re.kr (K.J.); berong35@kiom.re.kr (T.G.L.); 4 Korean Medicine Life Science, University of Science and Technology (UST), Daejeon 34054, Korea

**Keywords:** amygdalin, apricot kernel extract, keratoconjunctivitis sicca, urban particulate matter

## Abstract

Exposure to particulate matter is a risk factor for various ocular surface diseases, including keratoconjunctivitis sicca (KCS). In this study, we investigated the protective effects of apricot kernel extract (AKE) and its bioactive compound, amygdalin, on KCS induced by exposure to urban particulate matter (UPM). In the in vivo experiments, eye drops containing 0.5 mg/mL AKE (AKE-0.5) or 1 mg/mL AKE (AKE-1) were administered directly into the eyes of female rats after UPM exposure. Additionally, the effect of AKE and amygdalin on matrix metalloproteinases (MMPs) activity and the expressions of inflammatory factors, including tumor necrosis factor (TNF)-α and interleukin (IL)-6, was investigated in conjunctival epithelial cells in vitro. Topical administration of AKE-1 attenuated UPM exposure-induced reduction of tear secretion. Both AKE-0.5 and AKE-1 inhibited UPM exposure-induced corneal epithelial damage and irregularity. AKE also protected against UPM exposure-induced disruption of the mucin-4 layer on the ocular surface. In addition, AKE and amygdalin prevented UPM-induced activation of MMPs and upregulation of TNF-α and IL-6 in conjunctival epithelial cells. Therefore, AKE may have protective effects against UPM exposure-induced KCS via the inhibition of MMPs and inflammation. The pharmacological activities of AKE may be in part due to its bioactive compound, amygdalin.

## 1. Introduction

Keratoconjunctivitis sicca (KCS) is a common ophthalmological disease that results in symptoms of discomfort and the disturbance of vision [1,2]. KCS arises as a result of a disturbance of the ocular surface system, a complex entity that conceptually results from functional integration of its anatomical components (conjunctival epithelium, limbal epithelium, corneal epithelium, glandular epithelia, and tear film) with the adjacent structures (vasculature, nerves, eyelids, eyelashes, lacrimal glands, Meibomian glands, and nasolacrimal duct) [3]. More than 30% of the global population across age groups has the symptoms of KCS [1], and patients with KCS experience a lower quality of life [4].

Particulate matter is composed of solid and liquid particles, particularly those present in ambient air [5], and is a cause of air pollution that increases the public health risk [6]. Many studies have associated exposure to urban particulate matter (UPM) with respiratory diseases [7,8,9]. Furthermore, UPM is reported to play a role in promoting oxidative and inflammatory effects in various cells [6] and in inducing corneal cell apoptosis and inflammation [10]. However, few studies have focused on eye diseases, such as KCS, that are caused as a result of UPM exposure. The use of artificial tears is the treatment of choice for KCS; however, this only partially replenishes the aqueous tear fluid compartments [11], necessitating multiple topical applications. 

The apricot kernel is the seed of *Prunus armeniaca* L. (Prunus, apricot) [12], which is mainly cultivated in Asian countries, including China, Japan, and Korea [11]. Traditionally, the apricot kernel has been used as a herbal medicine for asthma, bronchitis, nausea, and constipation [13]. Apricot kernel oil has protective effects in the myocardium against ischemia–reperfusion injury [14]. In addition, the apricot kernel has been used to treat several skin diseases, such as furuncle, acne vulgaris, and dandruff [15], and in cosmetics to nourish, moisturize, and lubricate the skin. These pharmacological effects of the apricot kernel result from its anti-oxidant, anti-microbial [16], and anti-inflammatory [12] activities. Therefore, it is hypothesized that the use of apricot kernel may help ameliorate KCS. In this study, we investigated the effects of apricot kernel extract (AKE) on UPM-induced KCS and its mechanism. 

## 2. Results

### 2.1. HPLC Analysis of AKE

AKE quality was verified using a high-performance liquid chromatography (HPLC)-based method, and AKE was characterized by analysis of its major constituent, amygdalin. The concentration of amygdalin in AKE was 129.34 ± 0.99 mg/g.

### 2.2. Effects of AKE on Tear Secretion

**Figure 1:**
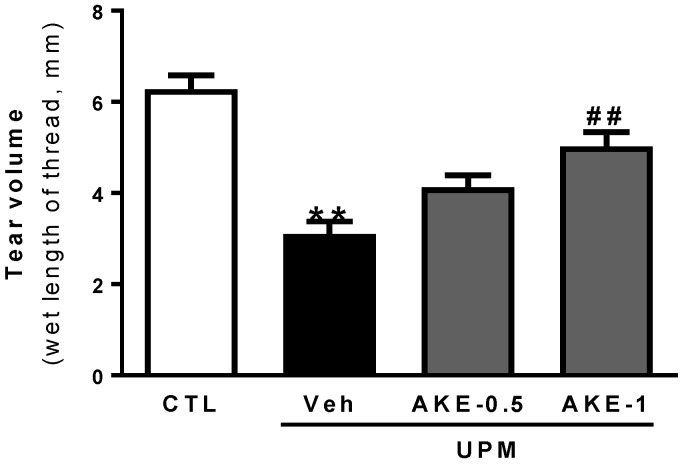
Effects of AKE on aqueous tear secretion in the UPM exposure-induced KCS model. The tear volume is expressed as the length of thread (mm) that turned red from the tear fluid. Control group: CTL; vehicle group: Veh; 0.5 mg/mL AKE-treated group: AKE-0.5; and 1 mg/mL AKE-treated group: AKE-1. Data shown are mean ± standard error of the mean (n ≥ 8). ** *p* < 0.01 vs. CTL, ## *p* < 0.01 vs. Veh.

As shown in Figure 1, tear volume, as measured by the phenol red thread tear test, decreased in the vehicle group (3.1 ± 0.28 mm) compared to that in the control group (6.3 ± 0.31 mm). The decrease in tear volume induced by UPM exposure was significantly reversed by topical administration of 1 mg/mL AKE (5.0 ± 0.31 mm), but not by topical administration of 0.5 mg/mL AKE (4.1 ± 0.26 mm).

### 2.3. Effects of AKE on Corneal Epithelial Damage

**Figure 2:**
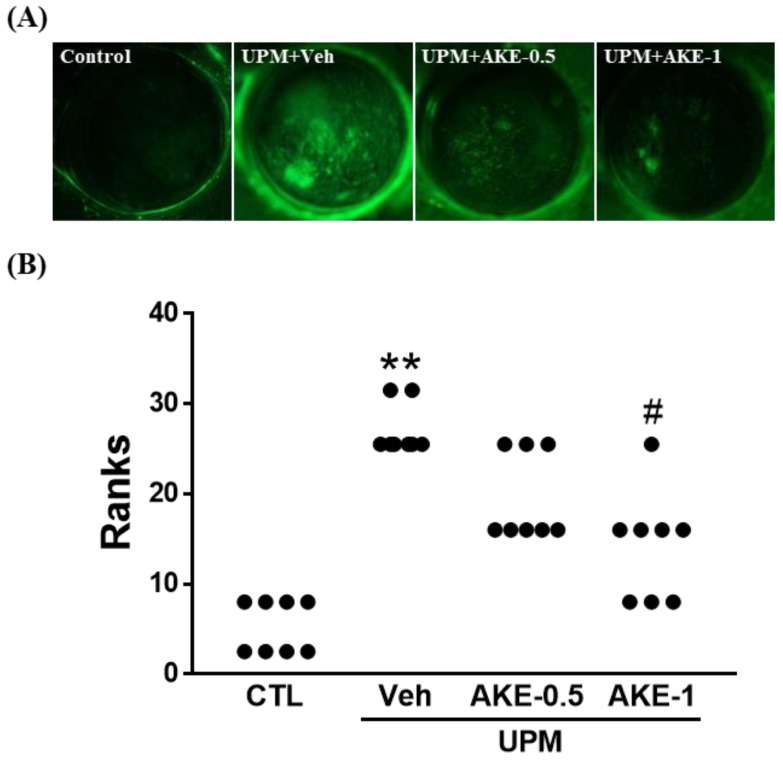
Effects of AKE on corneal epithelial damage in the UPM exposure-induced KCS model. (**A**) Representative images of corneal fluorescein. (**B**) Quantitative analysis of the corneal fluorescein staining score. Control group: CTL; vehicle group: Veh; 0.5 mg/mL AKE-treated group: AKE-0.5; and 1 mg/mL AKE-treated group: AKE-1. Data were presented as a median score using a ranks analysis (n ≥ 8). ** *p* < 0.01 vs. CTL, # *p* < 0.05 vs. Veh.

The corneal fluorescein staining score was used to evaluate the effects of AKE on corneal epithelial damage. The more corneal epithelium is damaged, the more fluorescein is observed. Corneal fluorescein was greater in the vehicle group than in the control group. Corneal fluorescein in the AKE-0.5 group and AKE-1 group was less than that in the vehicle group (Figure 2A). When corneal fluorescein staining was scored (Figure 2B), the corneal fluorescein staining score in the vehicle group (3.3 ± 0.16, mean ranks: 27.0) was significantly higher than that in the control group (0.5 ± 0.19, mean ranks: 5.3), and this increase was significantly attenuated by the administration of 1 mg/mL AKE (1.8 ± 0.25, mean ranks: 14.2), but not 0.5 mg/mL AKE (2.4 ± 0.18, mean ranks: 19.6).

### 2.4. Effects of AKE on Corneal Irregularity

**Figure 3:**
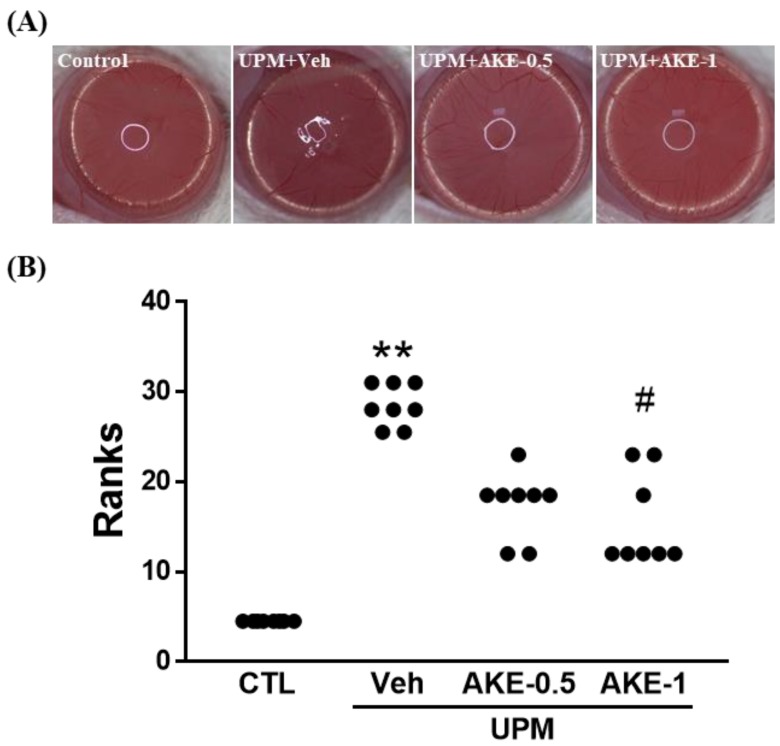
Effects of AKE on corneal irregularity in the UPM exposure-induced KCS model. (**A**) Representative reflected images of a white ring from the fiber-optic ring illuminator of the stereomicroscope. (**B**) Quantitative analysis of the corneal irregularity score. Control group: CTL; vehicle group: Veh; 0.5 mg/mL AKE-treated group: AKE-0.5; and 1 mg/mL AKE-treated group: AKE-1. Data were presented as a median score using a ranks analysis (n ≥ 8). ** *p* < 0.01 vs. CTL, # *p* < 0.05 vs. Veh.

The distortion of reflected light on the corneal surface can be regarded as a change in the stability of the tear film. As shown in Figure 3A, the circular shape in the cornea was maintained in the control group, but was severely broken in the vehicle group. This breakage was reduced by treatment with AKE (0.5 mg/mL and 1 mg/mL). To obtain quantitative results from pictures, the corneal irregularity score was assessed (Figure 3B). The corneal irregularity score was significantly increased in the vehicle group (3.6 ± 0.15, mean ranks: 28.5) compared with that in the control group (0.0 ± 0.00, mean ranks: 4.5). Administration of 1 mg/mL AKE significantly attenuated this UPM exposure-caused increase (1.5 ± 0.25, mean ranks: 15.6).

### 2.5. Effects of AKE on Mucin Integrity

**Figure 4:**
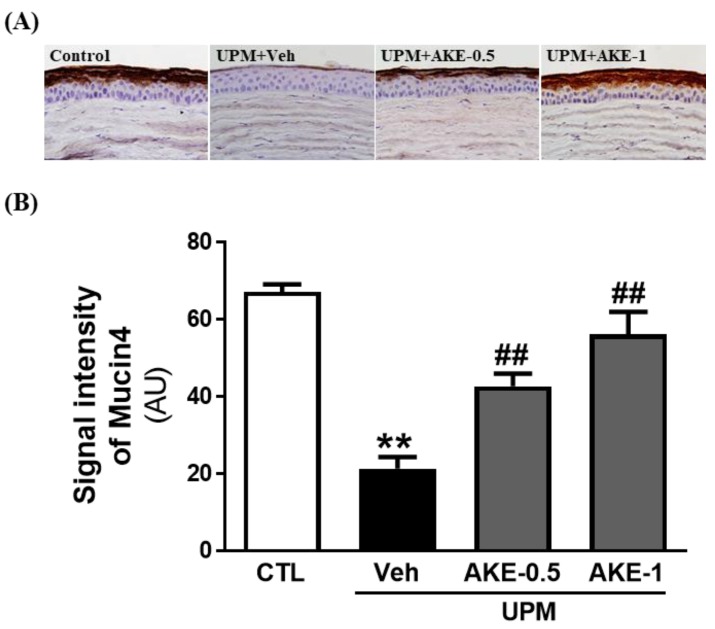
Effects of AKE on mucin-4 integrity in the UPM exposure-induced KCS model. (**A**) Representative images of mucin-4. (**B**) Quantitative analysis of the signal intensity of mucin-4. Control group: CTL; vehicle group: Veh; 0.5 mg/mL AKE-treated group: AKE-0.5; and 1 mg/mL AKE-treated group: AKE-1. Data shown are mean ± standard error of the mean (n ≥ 8). ** *p* < 0.01 vs. CTL, ## *p* < 0.01 vs. Veh.

We evaluated changes in the mucin-4 layer of the ocular surface by performing immunohistochemistry for mucin-4 in ocular surface sections. The mucin-4 layer in the ocular surface was disrupted in the vehicle group (21.4 ± 3.05 AU) compared to that in the control group (67.2 ± 1.94 AU), and this disruption was drastically reversed in both the AKE-0.5 and AKE-1 groups (42.8 ± 3.26 AU and 56.2 ± 5.81 AU, respectively) in a dose-dependent manner (Figure 4). 

### 2.6. Effects of AKE and Amygdalin on Matrix Metalloproteinases (MMP)

**Figure 5:**
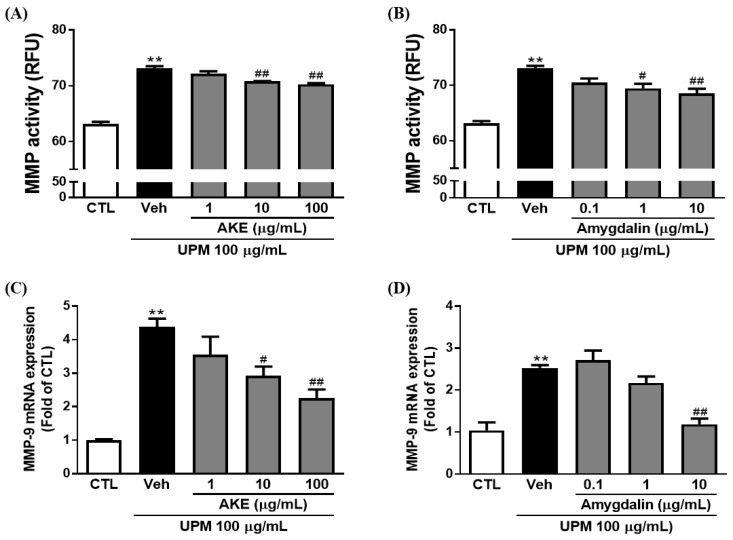
Effects of AKE on matrix metalloproteinase induced by UPM in the conjunctival epithelial cells. Conjunctival cells were treated with AKE or amygdalin 1 h before UPM exposure. (**A**) and (**B**) Activity of matrix metalloproteinase (MMP). (**C**) and (**D**) mRNA expression of MMP-9. Control group: CTL; vehicle group: Veh; AKE-treated group: AKE; urban particulate matter-treated group: UPM. Data shown are mean ± standard error of the mean (n ≥ 3). ** *p* < 0.01 vs. CTL, # *p* < 0.05 or ## *p* < 0.01 vs. Veh.

The effects of AKE and amygdalin on MMP activity and mRNA expression were investigated in conjunctival epithelial cells. MMP activity was elevated to 73.1 ± 0.41 RFU by 100 μg/mL UPM exposure, and 10 and 100 μg/mL AKE significantly reduced the elevation of MMP activity (70.8 ± 0.06 RFU and 70.3 ± 0.23 RFU, respectively) (Figure 5A). In addition, mRNA expression of MMP-9 was increased by 100 μg/mL UPM exposure (4.4 ± 0.24 fold), and this was significantly recovered by 10 and 100 μg/mL AKE (2.9 ± 0.27 fold and 2.3 ± 0.26 fold, respectively; Figure 5C).

In our previous study [11], we reported that 1 g of AKE contains 127.34 ± 0.99 mg of amygdalin. To investigate whether amygdalin acts as the bioactive component of AKE, we treated conjunctival epithelial cells with 0.1, 1, and 10 μg/mL of amygdalin. Consistent with the results obtained with AKE, as shown in Figure 5B, 1 μg/mL and 10 μg/mL of amygdalin (69.5 ± 0.80 RFU and 68.5 ± 0.86 RFU, respectively) significantly prevented the activation of MMP induced by UPM exposure (73.1 ± 0.41 RFU). In addition, 10 μg/mL of amygdalin (1.2 ± 0.14 fold) significantly inhibited the mRNA expression of MMP-9 induced by UPM exposure (2.5 ± 0.07 fold; Figure 5D). These findings reveal that amygdalin has a protective effect on MMP activation and mRNA expression as the bioactive component of AKE.

### 2.7. Effects of AKE and Amygdalin on Inflammation 

**Figure 6:**
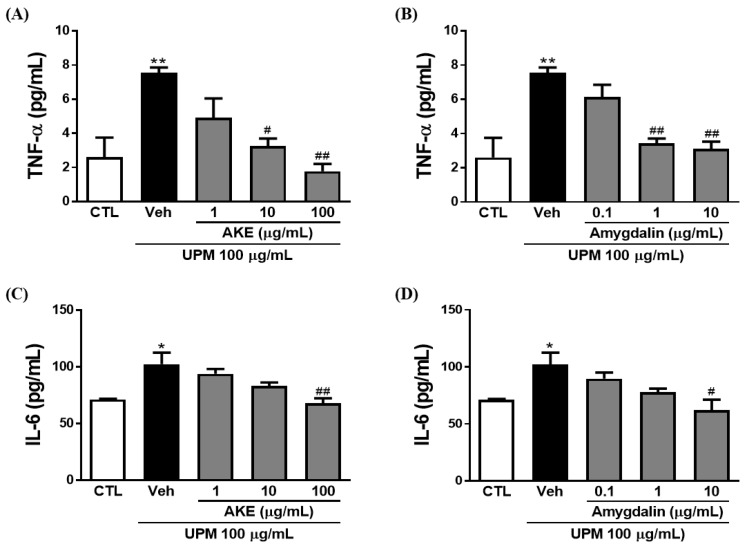

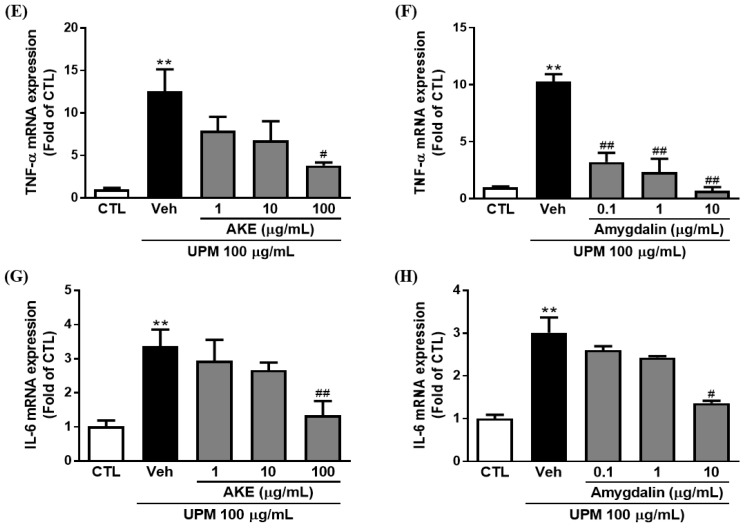
Effects of AKE on inflammation induced by UPM in conjunctival epithelial cells. Conjunctival cells were treated with AKE or amygdalin 1 h before UPM exposure. (**A**–**D**) The amount of TNF-α or IL-6 released. (**E**–**H**) mRNA expression of TNF-α or IL-6. Control group: CTL; vehicle group: Veh; apricot kernel extract-treated group: AKE; urban particulate matter-treated group: UPM. Data shown are mean ± standard error of the mean (n ≥ 3). * *p* < 0.05 or ** *p* < 0.01 vs. CTL, # *p* < 0.05 or ## *p* < 0.01 vs. Veh.

To investigate the effects of AKE and amygdalin on inflammatory factors, including tumor necrosis factor (TNF)-α and interleukin (IL)-6, enzyme-linked immunosorbent assay (ELISA) and quantitative polymerase chain reaction (qPCR) were performed in conjunctival epithelial cells. The release of TNF-α was increased by 100 μg/mL UPM exposure (7.5 ± 1.15 pg/mL). These increments of release were significantly decreased by 10 and 100 μg/mL AKE (3.3 ± 0.44 pg/mL and 1.8 ± 0.44 pg/mL, respectively) and 1 and 10 μg/mL amygdalin (3.4 ± 0.28 pg/mL and 3.1 ± 0.44 pg/mL, respectively) (Figure 6A,B). In addition, mRNA expression of TNF-α was increased by 100 μg/mL UPM exposure (12.6 ± 2.57 fold and 10.3 ± 0.67 fold); this was significantly recovered by 100 μg/mL AKE (3.8 ± 0.37 fold) and 0.1, 1, and 10 μg/mL amygdalin (3.2 ± 0.80 fold, 2.3 ± 1.15 fold, and 0.69 ± 0.32 fold, respectively; Figure 6E,F). 

Consistent with the results of the TNF-α, IL-6 was significantly released from conjunctival epithelial cells by UPM exposure (102.5 ± 10.06 pg/mL). This increased release of IL-6 induced by UPM exposure was significantly reduced by 100 μg/mL AKE (67.9 ± 4.61 pg/mL) and 10 μg/mL amygdalin (61.9 ± 9.47 pg/mL; Figure 6C,D). As shown in Figure 6G, the UPM-treated group showed greater IL-6 mRNA expression (3.4 ± 0.48 fold) than the control group. AKE at 100 μg/mL decreased IL-6 mRNA expression (1.3 ± 0.42 fold). The increased IL-6 mRNA expression induced by UPM exposure (3.0 ± 0.36 fold) was also significantly reduced by 10 μg/mL amygdalin (1.4 ± 0.05 fold).

## 3. Discussion

Exposure to particulate matter is a serious public health issue worldwide. Recently, many studies have differentiated particulate matter according to its median aerodynamic diameter, i.e., ultrafine, fine, and coarse particles [17], with a maximum diameter of 0.1 µm (PM0.1), 2.5 um (PM2.5), and 10 µm (PM10), respectively. Of these particles, the coarse NIST Standard Reference Material, UPM (SRM 1648a), has been used to study the biological effects of particulate matter [18,19,20,21]. Therefore, we used UPM in the present study. 

Because the eye is at high risk of exposure to particulate matter, the incidence of related eye diseases, including KCS, is high [1]. KCS is mainly caused by an impairment of the tear film, which causes a reduction in tear production and stability, and damage to the epithelium in the cornea and conjunctiva [22]. When tear production is decreased, tears no longer protect the ocular surface, resulting in KCS [22]. In addition, damage to the epithelium in the cornea and conjunctiva leads to barrier dysfunction, which is ascribed to KCS. In the present study, the 5-day UPM exposure decreased tear secretion and increased corneal epithelium damage. Topical administration of AKE extract reversed the reduction in tear secretion and ameliorated the corneal epithelium damage induced by UPM exposure, suggesting that AKE extract is useful for improving KCS induced by UPM exposure.

The corneal epithelial damage and irregularity are affected by MMPs released from the ocular surface, including the corneal and conjunctival epithelium [23]. Among MMPs, MMP-9 has a critical role in disrupting the epithelial barrier function [24,25]. In this study, our results show that mRNA expression and activity of MMP-9 were elevated in the conjunctival epithelium with UPM exposure; these were inhibited by AKE and amygdalin. These results suggest that AKE and amygdalin may regulate the barrier function by inhibiting the MMPs.

Tear film stability is important for maintaining ocular surface homeostasis, whose disruption is related to dry eye syndrome (DES), including KCS [22]. The ocular surface, including the cornea and conjunctiva, is covered with a thin tear film [26] composed of three layers [27]. The first and outermost layer of the tear film is an oily (lipid) component that maintains surface tension, viscosity, and elasticity [28]. The middle layer of the tear film is a watery (aqueous) component containing cytokines, immunoglobulins, and salts, and it serves to maintain tear osmolarity [29]. The innermost layer of the tear film is mucous-like (mucin) and acts as a hydrophilic barrier for eye protection [30]. The mucin layer is composed of three types of mucins categorized by their amino acid sequences: Gel-forming (mucin-2, 5AC, 5B, 6, and 19), soluble (mucin-7 and 9), and membrane-associated mucins (MAM) (mucin-1, 3A, 3B, 4, 12, 13, 15, 16, 17, 20, and 21) [31]. These mucins play an important role in maintaining a healthy tear film and contribute to homeostasis on the ocular surface [30]; the disruption of these mucins can lead to DES. Among MAM, especially, mucin 1, 4, and 16 were important molecules to ensure MAM barrier functionality. Although mucin-1 is the best biomarker of DES, there have been conflicting findings about the expression of mucin-1 in DES [32]. Corrales et al. [31] and Imbert et al. [33] reported that MUC-1 mRNA and protein expression was decreased in DES. In contrast, Gipson et al. [34] and Caffery et al. [35] reported that MUC-1 mRNA and protein expression was increased in DES. As mucin-4 is the second best biomarker after mucin-1 [32], it was an important classification of healthy patients. Previous studies indicated that mucin-4 is well associated with temporal lid-parallel conjunctival folds and lid-wiper epitheliopathy, which are clinical markers in DES-patients [36]. The level of mucin-4 is increased in the dry eye-induced ocular inflammatory environment to maintain a defense mechanism and recompense the loss of other mucins in the ocular surface [37]. Therefore, it can be suggested that the decrease in mucin-4 can accelerate the aggravation of ocular surface integrity. We evaluated changes in the mucin-4 layer in this study. The mucin-4 layer in the cornea was disrupted by UPM exposure, and this disruption was reversed by AKE treatment, indicating that AKE helps prevent disruption of the mucin-4 layer in the ocular surface.

The regulation of mucin on the ocular surface is important for the barrier function of the conjunctival epithelium, as with the corneal epithelium [28]. In KCS, the inflammatory factors, including IL-6 and TNF-α, in tears may regulate membrane-associated mucins expressed in the conjunctival and corneal epithelial cells [38]. In the present study, TNF-α and IL-6 were released in the conjunctival epithelium due to UPM exposure, and these were inhibited by AKE and amygdalin. These results suggest that AKE may regulate mucin by inhibiting inflammatory factors.

Although there are no reports about the effects of apricot kernel or its components on ocular surface except our previous study [11], amygdalin, a major component of AKE, elicits the production of gastric mucosal nitric oxide [39]. Nitric oxide induces mucin secretion from goblet cells [40]; thus, amygdalin has been reported to cause mucin secretion and maintain gastric mucosal integrity in an experimental gastric ulcer model [39]. In this study, amygdalin inhibited the UPM exposure-induced mRNA expression and release of MMP-9 and other inflammatory factors, including IL-6 and TNF-α. Therefore, the effect of AKE may be due to its bioactive compound, amygdalin.

The apricot kernel has been traditionally used for treatment of asthma, nausea, bronchitis, and so on. Although it has been mainly used for oral administration, ophthalmic drops containing AKE had protective effects against UPM-induced KCS in this study. Therefore, we suggest that AKE may be clinically used not after, but before or during, the onset of DES due to particulate matter. However, a study of the efficacy and safety of human use in clinical trials is necessary, since this study confirmed the efficacy in animal models.

In conclusion, our findings suggest that AKE may prevent UPM exposure-induced KCS, by increasing tear volume, inhibiting damage to the corneal epithelium, decreasing corneal surface irregularity, and attenuating disruption of the mucin-4 layer by inhibiting MMP-9, IL-6, and TNF-α. Therefore, AKE may have potential as a novel topical treatment for KCS due to particulate matter.

## 4. Materials and Methods

### 4.1. Materials 

UPM was purchased from Sigma-Aldrich (St. Louis, MO, USA; National Institute of Standards and Technology SRM 1648a).

### 4.2. Preparation of AKE 

AKE was obtained from the Korea Plant Extract Bank. The AKE preparation method has been described previously [11]. Briefly, seed kernel of *Prunus armeniaca* (500 g) was boiled for 2.5 h at 100 °C with distilled water (1.0 L) after drying and grinding. Then, the extract was concentrated using a freeze-drying method. AKE was deposited at the Herbarium of Korea Institute of Oriental Medicine (Daejeon, Korea). The contents of the major component in the AKE was determined by HPLC using amygdalin (Sigma-Aldrich, St. Louis, MO, USA) as the reference compound. The HPLC chromatograph of AKE has been previously reported [11]. 

### 4.3. Animals and Experimental Design 

The animal experiments were conducted according to a procedure approved by our Institutional Animal Care and Use Committee (IACUC approval No. 15-101). Female Sprague Dawley (SD) rats (6 weeks old) were purchased from Orient Bio (Seongnam, Korea). After accommodation for a week, the rats were randomly assigned to four groups: (1) Control group, (2) vehicle group, (3) 0.5 mg/mL AKE-treated group (AKE-0.5), and (4) 1 mg/mL AKE-treated group (AKE-1). KCS induction using UPM was performed by modification of procedures described previously [10]. Briefly, to induce KCS, 20 µL of UPM (20 mg/mL; dissolved in saline; Sigma-Aldrich) was topically administered onto the eyes of rats in the vehicle group, AKE-0.5 group, and AKE-1 group except the control group (saline-treated) thrice a day for 5 days according to the preliminary results (Figure S1). After the 5-day UPM treatment, all eyes in each group were topically exposed to 20 µL of saline or AKE (0.5 or 1 mg/mL) once per day for 5 days. The osmolality of the ophthalmic solution containing AKE was 290 mOsmol/kg. Side effects were not observed after the topical application of AKE.

### 4.4. Phenol Red Thread Tear Test

The phenol red thread tear test was performed as previously described [11]. Briefly, phenol red-impregnated cotton threads (Zone Quick; FCI Ophthalmics, Pembroke, MA, USA) were placed into the lateral canthus in the lower eyelid for 1 min. The tear volume was measured as the length of thread that turned red due to tear fluid and was expressed in millimeters (mm). 

### 4.5. Fluorescein Staining Score

Fluorescein staining was performed as previously described [11]. Briefly, fluorescein staining on the cornea was detected using a slit-lamp biomicroscope (Kowa Company Ltd., Nagoya, Japan) under cobalt blue light at 3 min after administration of 10 µL 1% fluorescein (Sigma-Aldrich) into the lateral conjunctival sac. The corneal fluorescein staining was scored as follows: 0, no spot; 1, ≤ 30 separate spots; 2, ≥ 30 separate spots in some area; 3, many separate spots without a closed spot in the overall area; 4, many separate spots with closed spots in the overall area.

### 4.6. Irregularity Score

The lines on the corneal surface reflected by a ring-shaped light from the fiber-optic ring illuminator of a stereomicroscope (SZ51; Olympus, Tokyo, Japan) were captured using a DP21 digital camera (Olympus). The corneal irregularity was scored as follows: 0, no distortion; 1, distortion in one quadrant; 2, distortion in two quadrants; 3, distortion in three quadrants; 4, distortion in all four quadrants; and 5, severe distortion in which no ring could be recognized.

### 4.7. Immunohistochemistry

At necropsy, eyes were removed from the rats. The paraffin-embedded eye tissue was sectioned. Sections on slide were labeled with an LSAB kit (DAKO, Santa Clara, CA, USA) and anti-mucin-4 antibody (Invitrogen, Cat# PA5-23077, 1:1000). Then, sections were visualized with a DAB substrate kit (DAKO). All sections were observed under a fluorescence microscope (Olympus). Signal intensity of mucin-4 was measured using Image J software v.1.51j8 (NIH, Bethesda, MD, USA).

### 4.8. Cell Culture

Conjunctival epithelial cells were obtained from the Korean Cell Line Bank (Seoul, Korea) and maintained at 37 °C in RPMI 1640 containing 10% fetal bovine serum in a 5% CO_2_ incubator. Cells were treated with AKE (1, 10, or 100 μg/mL) and amygdalin (0.1, 1, or 10 μg/mL) 1 h before 100 μg/mL UPM exposure treatment. 

### 4.9. Detection of MMP Activity 

At 24 h after UPM exposure treatment, MMP activity in the cell culture media was detected using a MMP activity assay kit (Abcam, Cambridge, UK). The fluorescence intensity was measured using a microplate reader (BioTek Instruments, Inc., Winooski, VT, USA) at 490 nm/525 nm (excitation/emission).

### 4.10. Detection of Inflammatory Factors 

At 24 h after UPM treatment, cell culture media was collected and centrifuged to eliminate the cell debris. Inflammatory factors, such as IL-1β, IL-6, and TNF-α, were detected in the supernatant using an ELISA kit for each (Abcam). The fluorescence intensity was measured using a microplate reader (BioTek Instruments, Inc.) at 490 nm/525 nm (excitation/emission).

### 4.11. Quantitative Polymerase Chain Reaction (qPCR) 

At 18 h after UPM exposure treatment, the RNA obtained using the RNeasy Plus Mini Kit (QIAGEN, Hilden, Germany) was reverse transcribed to cDNA using an iScript cDNA Synthesis Kit (Bio-Rad Laboratories, Inc., Hercules, CA, USA). Then, qPCR was performed using SYBR Green Supermix (Bio-Rad Laboratories, Inc.). The primers were also purchased from Bio-Rad Laboratories, Inc. The results were normalized to glyceraldehyde 3-phosphate dehydrogenase (GAPDH).

### 4.12. Statistical Analysis

Statistical analysis was performed using one-way analysis of variance (ANOVA) followed by Tukey’s multiple comparison test between groups using Prism 7.0 software (GraphPad, San Diego, CA, USA). The data of the fluorescein staining score and irregularity score were analyzed using Kruskal Wallis non-parametric ANOVA with Dunn’s multiple comparisons test. *p* < 0.05 was considered statistically significant.

## Supplementary Materials

The supplementary materials are available online.
