# Supplementary material for: Apricot Kernel Extract and Amygdalin Inhibit Urban Particulate Matter-Induced Keratoconjunctivitis Sicca
Source: Molecules. 2019 Feb 12;24(3):650. doi: 10.3390/molecules24030650 (PMC6384987; doi:10.3390/molecules24030650)
Supplement: Supplementary file 1 [file molecules-24-00650-s001.pdf]

molecules

Article

Apricot Kernel Extract and Amygdalin Inhibit Urban Particulate Matter-Induced Keratoconjunctivitis Sicca

Soo-Wang Hyun [1,†], Junghyun Kim [2,†], Bongkyun Park [3], Kyuhyung Jo [3], Tae Gu Lee [3], Jin Sook Kim [1] and Chan-Sik Kim [3,4,*]

Supplementary Materials:

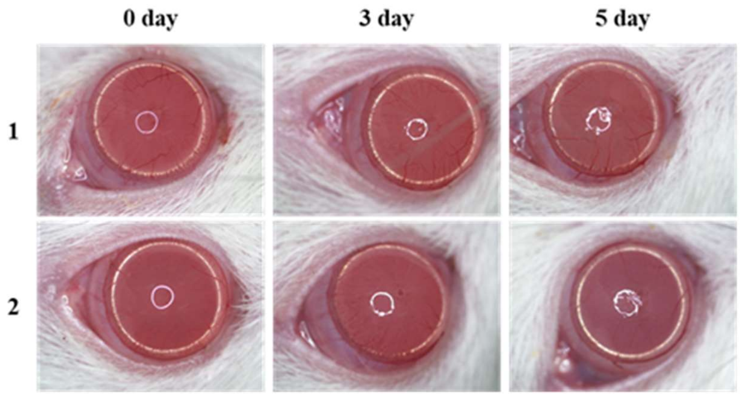

Figure S1. The time series of change in corneal irregularity. Representative reflected images of a white ring from the fiber-optic ring illuminator of the stereomicroscope. The images were taken o, 3, and 5 days after exposure to urban particulate matter.
